# A Review of Numerical Simulation of Laser–Arc Hybrid Welding

**DOI:** 10.3390/ma16093561

**Published:** 2023-05-06

**Authors:** Zhaoyang Wang, Mengcheng Gong, Longzao Zhou, Ming Gao

**Affiliations:** 1 Wuhan National Laboratory for Optoelectronics (WNLO), Huazhong University of Science and Technology, Wuhan 430074, China; 2 School of Materials Science and Engineering, Huazhong University of Science and Technology, Wuhan 430074, China

**Keywords:** numerical simulation, laser–arc, interaction, welding

## Abstract

Laser–arc hybrid welding (LAHW) is known to achieve more stable processes, better mechanical properties, and greater adaptability through the synergy of a laser and an arc. Numerical simulations play a crucial role in deepening our understanding of this interaction mechanism. In this paper, we review the current work on numerical simulations of LAHW, including heat source selection laws, temperature field, flow field, and stress field results. We also discuss the influence of laser–arc interaction on weld defects and mechanical properties and provide suggestions for the development of numerical simulations of LAHW.

## 1. Introduction

International Welding Commission defines welding standards as a process involving the application of heat or pressure (often localized) to join parts together in a non-detachable manner, either through the addition or absence of filler material or by overlaying the substrate surface [1]. Theodore Maiman developed the world’s first ruby laser in 1960, which attracted a lot of research interest due to its high brightness, directivity, monochromaticity, and coherence. However, it was not until the advent of high-power lasers and special optical fibers that lasers became widely used in the field of welding [2]. The laser method is based on Einstein’s theory of stimulated radiation. The process involves exciting electrons to high energy levels via the pump, the pump substance, and the resonant cavity. These electrons transition back to lower energy levels and release photons, which are then amplified to create a laser [3]. Compared to arc welding, laser welding has irreplaceable advantages in thick plate welding due to its high efficiency and penetration. However, it also has limitations, such as high assembly requirements, easy generation of porosity and cracks, and low clearance tolerance, which hinder its practical application [4].

Around 1980, Steen [5] introduced the concept of hybrid welding for the first time, and since then hybrid welding has been widely studied around the world. Laser–arc hybrid welding, using the combined heat source of laser and arc, enables high-speed welding with high filling efficiency, better shape, fewer weld defects, and excellent mechanical properties. It is widely used in fields such as transportation and aerospace [6,7,8,9,10]. Although hybrid welding has many advantages, it is difficult to achieve high-quality welds in practical applications due to the complex interaction mechanism of laser and arc in space–time. The simplest and most universal method to achieve optimal welding process and results are by adjusting welding parameters to observe and macroeconomically regulate and control the optimal process interval. This approach results in fewer defects and high-performance welds [11,12,13,14]. However, macroeconomic regulation and control based on parameter optimization cannot solve the problem in essence and cannot cope with the intense interaction between laser and material in welding. With the development of physics and technology, X-ray detection, high-speed cameras, and so on appear one after another. People can observe and verify by any means (such as high-speed photography, spectral analysis, and X-ray observation, etc.), and the physical mechanism of laser–arc interaction is investigated [15,16,17,18]. However, the shortcomings of high preconditions, high resource consumption, and cumbersome steps have greatly slowed down the pace of scientific research. The emergence of numerical analysis provides a low-cost, high-efficiency means of selecting parameters, and the visual description of multi-physical fields in the welding process also provides a great help in completing the physical mechanism of laser–arc interaction.

While numerical simulation has been effective at predicting and verifying experiments, the advent of finite element analysis and computers brought about rapid development in this field [19]. The step-by-step method of numerical simulation allows for the gradual visualization of high-speed and violent reactions in laser–arc hybrid welding (LAHW), which meets the requirements of this process [20]. In the numerical simulation of welding, to explain the interaction mechanism between complex temperature fields and material, laser and arc heat sources are optimized to adapt to different welding environments [21,22,23,24], it has become a common phenomenon to adopt different heat source models for different emphasis. To save on computational costs and improve computational efficiency, the existing numerical simulation mainly focuses on the influence of the temperature field on the flow field and stress field [25,26,27]. The accuracy of the model has been evaluated by the temperature field profile and the law of flow field and stress field [28,29,30].

This paper discusses the effects of LAHW on the simulation results and the microstructure and properties of weld defects in three parts. The first part mainly describes the law of heat source selection, the second part mainly describes the change law of the multi-physical field in LAHW, and the third part mainly describes the law of its influence on the microstructure and properties of weld defects.

## 2. Selection of Laser and Arc Heat Source

**Figure 1:**
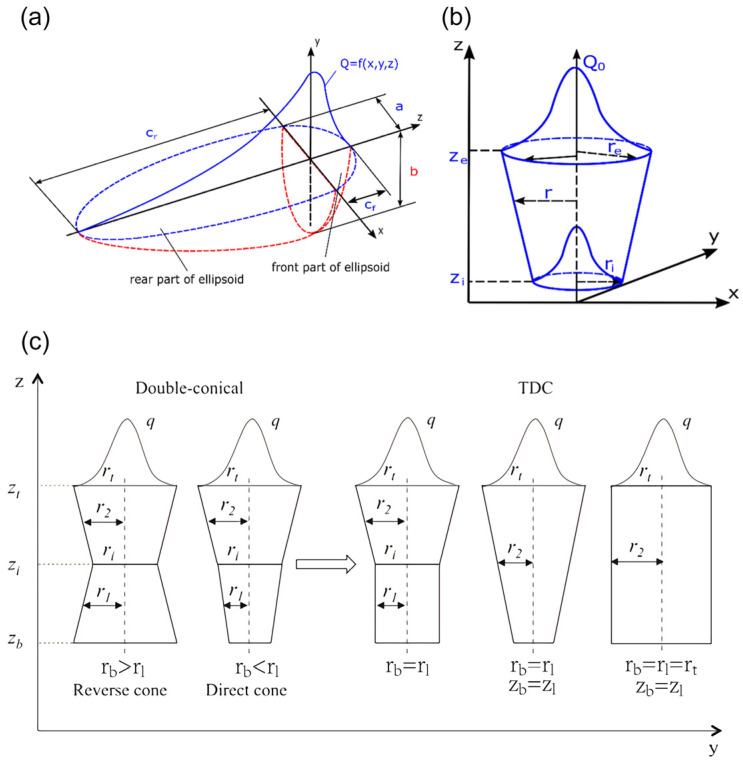
(**a**) double ellipsoid heat source; (**b**) Gauss heat source [44]; (**c**) variant Gaussian heat source [42].

The heat source equation, as a basic thermal physical condition in the numerical simulation of LAHW, has received much attention from researchers. The more mature heat source models are the double ellipsoid heat source, the Gaussian heat source, and the variant heat source based on these two types of heat sources [31,32,33], in which arc heat source is usually represented by a double ellipsoid heat source or surface heat source, and laser heat sources are often represented by Gaussian heat sources. The interaction between the heat source and the material in the numerical simulation of laser–arc welding is presented by the coupling between them, and its mechanism is explained. As shown in Figure 1a, the double ellipsoid heat source is mainly composed of two identical hemi-ellipsoids with different short axes on the long axis, and the heat source formula is shown in Formulas (1) and (2) [34]. As shown in Figure 1b, since the energy distribution of the laser spot conforms to the Gaussian distribution, the Gaussian heat source has been used as the main representation of the laser heat source in recent years. The main distribution pattern is shown in Formula (3) [35], and in the stress simulation, the main distribution pattern is shown in Formula (4).

Compared to arc heat sources, more work is required to improve the accuracy of numerical models for the laser heat sources. In laser deep penetration welding, the incident laser is repeatedly reflected and absorbed by the keyhole wall. In order to account for the effect of wall reflection and absorption, this effect has been introduced by changing the absorption efficiency of the material to the laser [36]. The formation of the keyhole is closely related to the recoil pressure of metal vapor, and the heat loss due to metal vapor evaporation must be taken into account [37]. For example, Li et al. [38] considered the external pressure, internal evaporation, and recoil pressure of metal vapor by introducing the Wilson equation and the gas–liquid equilibrium equation, the numerical simulation of molten pool flow in laser welding of the aluminum alloy under different pressure was realized. Cho et al. [39] used Flow-3D v.11.2 (2017) numerical simulation software to simulate the process of welding aluminum alloy with sinusoidal oscillating laser wire filler. By combining the Gauss heat source with the sinusoidal oscillating trajectory, the oscillating laser Gaussian heat source was realized. Under the conditions of recoil pressure Formula (5), fender reflection absorption and evaporation, and steam pressure, the numerical simulation of aluminum alloy wire-filled welding with sinusoidal oscillation track of laser heat source was completed. For example, Yu et al. [40] used a laser Gaussian heat source to realize the stress simulation of welding 5A90Al-Li alloy, and the experimental results have a high degree of coherence. Tsirkas et al. [41] realized the numerical simulation of CO_2_ laser welding of aircraft aluminum alloy components using numerical analysis software under the condition of introducing the “Life and Death Unit”.

As shown in Figure 1c, based on the Gaussian heat source, the shape and energy distribution of the laser heat source can be adjusted by changing the radius and energy distribution of the heat source at the top, middle, and bottom. The heat source formula is shown in Formulas (6) and (7). For example, Farrokhi et al. [42] introduced r_t_, r_b_, r_i_, z_b_, z_t_, z_i_ and other physical quantities into the numerical simulation of temperature–stress by changing the shape and energy distribution of the Gaussian heat source; thus, the error between the simulated weld and the actual shape was smaller and more accurate. Geng et al. [43] introduced the deformed Gaussian heat source into the numerical simulation of the temperature-flow field and obtained the hourglass-shaped keyhole and weld. It was concluded that Marangoni convection was mainly distributed near the keyhole and decreased with the increase in the distance from the keyhole.

(1) $$Q_{f}=\frac{6\sqrt{3}Ak_{f}\delta_{1}P}{c_{f}ba\pi \sqrt{\pi }}\exp\begin{array}{c} \left\{-3\left[\frac{{\left(x-x_{0}\right)}^{2}}{a^{2}}+\frac{{(y-y_{0})}^{2}}{b^{2}}+\frac{{\left(z-z_{0}\right)}^{2}}{c_{f}^{2}}\right]\right\}\\ -b\leq y\leq 0,z\geq z_{0} \end{array}$$

(2) $$Q_{r}=\frac{6\sqrt{3}Ak_{r}\delta_{2}P}{c_{r}ba\pi \sqrt{\pi }}\exp\begin{array}{c} \left\{-3\left[\frac{{\left(x-x_{0}\right)}^{2}}{a^{2}}+\frac{{\left(y-y_{0}\right)}^{2}}{b^{2}}+\frac{{\left(z-z_{0}\right)}^{2}}{c_{r}^{2}}\right]\right\}\\ -b\leq y\leq 0,z\leq z_{0} \end{array}$$

(3) $$Q=\frac{9Ak_{s}P}{R_{0}^{2}\pi \left(1-e^{-3}\right)}\exp\left\{-9\frac{\left[{\left(x-x_{0}\right)}^{2}+{\left(y-y_{0}\right)}^{2}\right]}{R_{0}^{2}\log\left[H/\left(z-z_{0}\right)\right]}\right\}$$

(4) $$Q=\frac{9Ak_{s}P}{R_{0}^{2}\pi H\left(1-e^{-3}\right)}\exp\left\{-9\frac{\left[{\left(x-x_{0}\right)}^{2}+{\left(y-y_{0}\right)}^{2}\right]}{R_{0}^{2}\log\left[H/\left(z-z_{0}\right)\right]}+\frac{{\left(z-z_{0}\right)}^{2}}{H^{2}}\right\}$$

(5) $$P_{r}≅0.54P_{0}exp(H_{lv}\frac{T-T_{g}}{RTT_{g}})$$

(6) $$Q_{l1}=\frac{9P_{l1}e^{3}}{\pi \left(e^{3}-1\right)\left(z_{i}-z_{b}\right)\left(r_{i}^{2}+r_{i}r_{b}+r_{b}^{2}\right)}\exp\left\{-3\frac{\left[{\left(x-x_{0}\right)}^{2}+{\left(y-y_{0}\right)}^{2}\right]}{r_{1}^{2}}\right\}$$

(7) $$Q_{l2}=\frac{9P_{l2}e^{3}}{\pi \left(e^{3}-1\right)\left(z_{t}-z_{i}\right)\left(r_{t}^{2}+r_{i}r_{t}+r_{i}^{2}\right)}\exp\left\{-3\frac{\left[{\left(x-x_{0}\right)}^{2}+{\left(y-y_{0}\right)}^{2}\right]}{r_{2}^{2}}\right\}$$

(8) $$\left\{\begin{array}{c} r_{1}\left(z\right)=r_{i}-\left(r_{i}-r_{b}\right)\frac{z_{i}-z}{z_{i}-z_{b}}\\ r_{2}\left(z\right)=r_{t}-\left(r_{t}-r_{i}\right)\frac{z_{t}-z_{1}}{z_{t}-z_{i}} \end{array}\right.$$

(9) $$\left\{\begin{array}{c} P_{l1}=\frac{z_{i}-z_{b}}{z_{t}-z_{b}}P\\ P_{l2}=\frac{z_{t}-z_{i}}{z_{t}-z_{b}}P \end{array}\right.$$

where a is the laser absorptivity, k_S_ is the ratio of laser energy to Gauss body heat source (k_S_ = 1 when there is only a Gauss body heat source), P is the laser power, R_0_ is the radius of the laser spot, H is the height of the heat source, (x_0_, y_0_, z_0_) is the center coordinate of the heat source, k_f,_ k_r_ is the ratio of laser energy to the cover heat source, δ_1_ is the ratio of the first half of the double ellipsoid heat source, δ_2_ is the ratio of the second half of the double ellipsoid heat source, and a, b, and c_f_, c_r_ are the parameters used to define the size and shape of the double ellipsoid. The parameters of r_t_, r_b_, r_i_, z_b_, z_t_ and z_i_ are shown in Figure 1c.

In the numerical simulation of laser–arc hybrid welding, researchers usually make assumptions about the model to reduce the difficulty of modeling and reduce the calculation time of the model. In fluid modeling, common assumptions are: (i) the molten metal is assumed to be incompressible Newtonian fluid; (ii) the deposition of laser heat is in conduction mode due to the high welding speed as well as high oscillating frequency of the laser beam; (iii) the small gap between the two sheets in the lap joint to help the aluminum vapor to escape is ignored in the simulation, since the gap has insignificant effects on the weld geometry and fluid flow in the weld pool; (iv) average current and voltage are used to calculate the arc power, and the pulse effect is neglected [36,37,38,45]. The common assumptions used in stress simulation are: (i) phenomena such as vaporization, ejection of material, circulation of the molten metal, formation of ions, etc., were left out of this work. (ii) Both the initial specimen temperature and the ambient temperature were 20 °C. (iii) The welded steel was considered a flat plate. (iv) The phase change and material flow in the welding zone is not considered. Heat source, heat transfer, and boundary condition of radiation and convection were considered to analyze the thermal data [40,41,46,47,48].

## 3. Influence of Laser–Arc Interaction on Multi-Physical Fields

When two kinds of heat sources (laser and arc heat source) act on the weld, they will have a great effect on the temperature field, increase the pool volume and surface area, promote the formation of a complex pool in the pool, and then affect the weld microstructure and mechanical properties. Additionally, the different coupling modes between the laser and the arc will have a great impact on the influence of multi-physics; for example, different coupling and spatial distribution, such as paraxial coupling, coaxial coupling, laser defocus, and the relative position of the laser and arc will have a great influence on multi-physics in LAHW.

**Figure 2:**
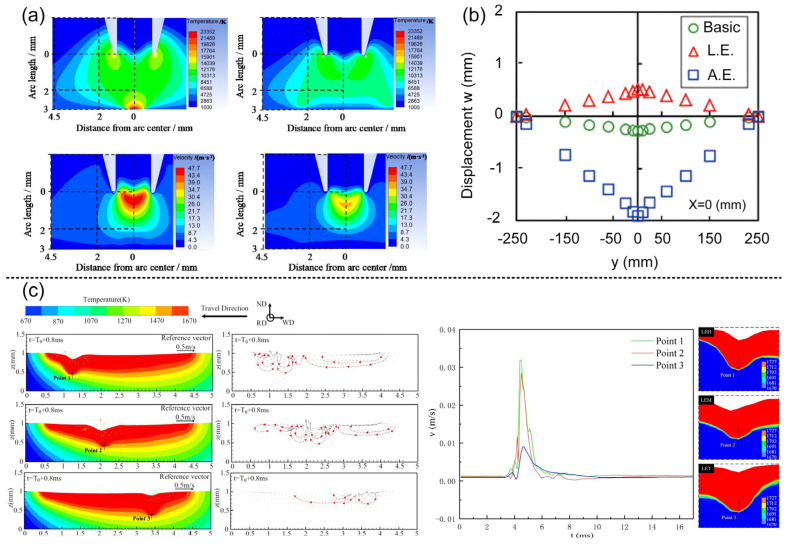
(**a**) Comparison of temperature field and velocity field in coaxial coupling and arc welding [49]; (**b**) influence of laser defocusing on deformation angle after welding [50]; (**c**) influence of the relative position of laser and arc on temperature field, flow field, and flow velocity [51].

In coaxial coupling, the energy density of the laser and arc will be superimposed on the same central point, and the interaction between heat and matter will undergo a series of different changes under the condition of high energy density, the most intuitive performance in the temperature field temperature gradient changes. The coaxial coupling of arc and laser not only increases the laser power at the center point but also increases the heating area around the center point. As shown in Figure 2, in the coaxial coupling [49], by comparing the temperature field and the melt flow field of arc welding, it can be found that the coaxial coupling is caused by the addition of a central laser heat source. In the paraxial coupling, the laser and arc are located at different center points and move to the welding direction at the same speed. The different heat source center points lead to an increase in the heating area and the sequence of the laser and arc heat source acting at the same point. Therefore, the relative position of the laser and arc has a great influence on the multi-physical fields and deformation of welding in paraxial recombination. Bakir et al. [32] found that the absolute value of laser defocusing was positively correlated with the number of weld cracks and concentrated at the root of the weld by adjusting the ratio of laser to arc heat source heat input. As shown in Figure 2b, Kim et al. [50] found that when arc heat input is dominant, the angular deformation is v-shaped, and when laser heat input is dominant, the angular deformation is inverted v-shaped; as shown in Figure 2c, it was found by Cai et al. [51] that the relative position of laser and arc can affect the velocity and direction of melt flow, and then affect the grain size and mechanical properties of the weld, the minimum grain size and the highest mechanical properties can be obtained via laser at the front of the arc region.

**Figure 3:**
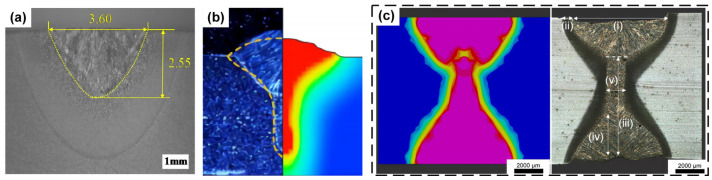
(**a**) coaxial coupling weld profile [52]; (**b**) paraxial coupling weld profile, light blue and red are experimental and simulated weld shapes [53]; (**c**) penetration weld profile [54]. (i) Weld width on the upper surface; (ii) HAZ; (iii) Weld width on the middle zone; (iv) laser zone penetration; (v) the lower surface width.

The different energy characteristics of laser and arc lead to the different shape characteristics of weld. The increase in arc current often leads to a faster increase in weld width than penetration, and the increase in laser power will have more advantages in the depth of penetration. The different ratio of laser to arc has great influence on the weld shape during laser–arc hybrid welding. As shown in Figure 3a, a low-power laser and high-current arc are common in the coaxial coupling, the weld shape is v-shaped, and the penetration depth increases with the increase in laser power. As shown in Figure 3b, in the paraxial coupling, the weld is wide and shallow like a bowl in the upper arc region, narrow and deep in the lower laser region, and the weld width decreases sharply as a thin neck because of the higher penetration of the laser relative to the arc. As shown in Figure 3c, when the laser power is further increased to penetration, the weld appearance is mainly an hourglass shape. At this time, a wider molten pool will appear at the bottom under the action of the surface tension of the molten pool and the backing force of the protective gas at the bottom and keep it steady.

## 4. Explaining the Mechanism of Laser–Arc Interaction

### 4.1. Defect Suppression

In LAHW, welding parameters (such as laser power, arc current, and wire spacing), heat source parameters, and process parameters (such as shielding gas flow rate and welding speed) have great influence on laser–arc interaction, and help to achieve the control of weld formation, welding process stability, and welding defects and other purposes [55,56,57]. Gas hole defect is the key research object of welding-related subjects because it can reduce the effective working section of the weld seam, lead to stress concentration, and then reduce the mechanical properties of the weld seam. The keyhole is easy to collapse in laser welding, so it is easy to form process-type pores. However, the temperature gradient of laser–arc hybrid welding decreases due to the addition of an arc heat source, which leads to the increase in the molten pool area and a longer solidification time. Gao et al. [58] found that AZ31B magnesium alloy can be undercut and blowhole defects can be suppressed by LAHW; by comparing the microstructures and mechanical properties of MIG and MIG-Laser-hybrid welding pure copper, Zhang et al. [12] found that hybrid welding can achieve narrower HAZ, finer grain structure, and higher conductivity. The fundamental reason for this that there is less porosity and impurity in the composite welding.

In laser–arc hybrid welding, the arc droplet will strike the surface of the molten pool and cause oscillation of the molten pool and keyhole, which easily forms humps and dent on the weld surface. At this point, the way the laser and the ARC are guided will have a more important effect on the impact force. Tang et al. [59] considered that the balance between gravity and surface tension of a molten pool is an important factor to influence the appearance of the hump; however, the arc-guided LAHW has a stronger restraining effect on the root hump defects because of its smaller droplet diameter and longer transition period. Zhang et al. [60] found that compared with arc welding, the spatter of LAHW is reduced, employing spectral analysis and high-speed photography of plasma plume in LAHW; moreover, the welding process is more stable, and the laser–arc interaction can promote the photon energy level transition to enhance the heat input, which results in a lower droplet transfer force. To clarify and supplement the mechanism of laser–arc interaction, researchers have conducted a lot of research using numerical analysis.

**Figure 4:**
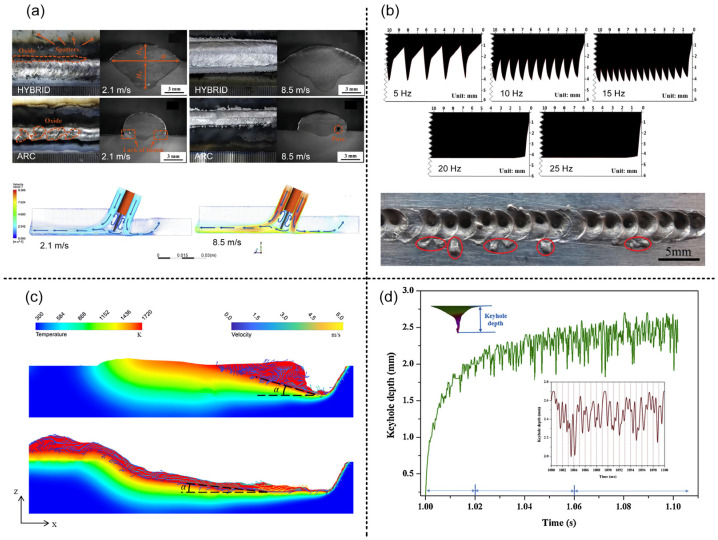
(**a**) the effect of gas flow rate on weld profile (top) and gas flow field (bottom) [64]; (**b**) the effect of pulse frequency on weld profile (top) and weld surface formation (bottom) under a high excitation current [65]; (**c**) the influence of filament spacing on arc plasma area [66]; (**d**) the variation rule of the keyhole in LAHW [68].

As an inert gas protective layer used to prevent metal oxidation in the molten pool, the related parameters affect the melting effect and even the depth of molten metal. For example, during the welding of carbon steel, carbon dioxide gas is mixed into the inert gas to achieve the effect of increasing penetration [61]. In laser welding, the plasma above the weld produces the absorption effect on the laser. The plasma is heated and excited to expand, which increases the absorption efficiency, and then forms positive feedback [62]. At this time, the protective gas not only plays the role of protecting the molten pool metal but also plays the role of blowing away the plasma above the weld, which plays a greater role in increasing the penetration of laser welding [63]. As shown in Figure 4a, Yang et al. [64] found that the effect of high shielding gas flow rate on LAHW was less than that of single-arc welding, and a high gas flow rate had a positive effect on the spread of molten metal. It is shown that the high-speed and high-pressure gas field was caused by the high shielding gas flow rate above the weld, and the plasma was easily affected by this field in arc welding. The explanation for this is that the effect of this field and the binding of the plasma were effectively restrained by the high-pressure steam ejected from the keyhole during the hybrid welding.

In laser–arc hybrid welding, the welding stability is the direct-viewing factor that affects the welding defects. In the welding dynamic stability stage, when the laser or the arc appears to be an out-of-cycle fluctuation, it will probably cause the welding process to destabilize. The stability of the laser keyhole and arc droplet transfer process affects the weld quality. A more stable droplet transfer process, such as jet transfer, reduces spatter and minimizes the impact on the molten pool. This process is crucial to maintaining the stability of the molten pool. As shown in Figure 4b, Huo et al. [65] performed numerical simulation of pulsed LAHW of magnesium alloy and found that the maximum energy efficiency can be achieved by keeping the pulse frequency at 20 Hz with the optimum range of laser excitation current being 150–175 A. If the pulse frequency is less than 20 Hz, the longitudinal profile of the weld will be discontinuous, and if the laser excitation current is too high, serious spatter will occur. As shown in Figure 4c, Xue et al. [66] discovered that compound welding effectively inhibits hump defects compared to laser welding. They explained that the TIG heat source introduces arc force and increases the area of the molten pool, which can be efficiently suppressed by high-speed melt flow in laser welding, leading to the formation of the hump.

In laser–arc hybrid welding, laser heating directly heats the arc as it passes through it [61]. Metal vapor ejected from the keyhole can alter the composition of the arc plasma and influence fluid flow and heat transfer [62]. The combined plasma above the weld absorbs the laser energy, which reduces the laser’s thermal efficiency [63]. The interaction between laser-induced metal vapor and arc plasma is affected by laser power, arc current, and the relative positions of laser and arc. Mu et al. [67] discovered through experiments and numerical simulations that high-speed melt flow in laser welding can be effectively inhibited. In LAHW, the arc tail oscillates at a frequency of 1–3 kHz, and the filament spacing affects the arc plasma area significantly. The high-speed metal vapor ejected from the laser keyhole exerts physical shielding, which compresses the arc plasma. The high-pressure metal vapor in the keyhole maintains the stability of the Keyhole, and the keyhole wall handles the equilibrium state of steam recoil pressure, surface tension, and gravity, etc. Each bulge is a potential cause of keyhole collapse, where the bulge is more susceptible to force imbalance, cutting off the keyhole cavity and creating bubbles of gas below. Finally, it is captured by the solidified molten pool and becomes a gas hole defect. The probability of keyhole collapse increases with the number of protrusions in the molten metal. As shown in Figure 4d, Chen et al. [68] observed that the oscillating frequency of the keyhole in hybrid welding is several kilohertz, and the keyhole change can be divided into three stages: initial establishment, rapid deployment, and oscillatory dynamic stability. The instability of the welding process primarily occurs during the stage of keyhole closing and re-opening. The formula for oscillation frequency is given $f_{k}={\left(\gamma /r^{3}\rho \right)}^{1/2}$, and the surface tension primarily causes keyhole instability [69].

Laser–arc hybrid welding can increase the likelihood of bubble overflow by increasing the molten pool’s area and solidification time, but it does not effectively suppress porosity in aluminum alloy welding. Keyhole collapse and arc droplet impact in high-power lasers still significantly influence welding spatter and hump. Unstable welding processes and welding defects affect the solidification and mechanical properties of welds, leading to coarse grain, variable element distribution, and decreased mechanical properties.

### 4.2. Stress and Mechanical Properties

Mechanical properties are key factors for assessing weld quality and are influenced by factors including weld microstructure, defects, and stresses [70,71,72]. Strengthening elements in welding, on one hand, play an important role in enhancing the properties of welds. For example, Si in Al alloy enhances the fluidity of the molten metal while also forming new compounds with the base metal, ultimately reinforcing the properties of the weld. Hao et al. [73] observed that Si and Sn in LAHW of pure copper improved the tensile strength and corrosion resistance but lowered electrical conductivity. They explained the promotion of columnar crystal growth in the fusion zone by Si and Sn, thereby enhancing tensile properties using the solid solution strengthening mechanism. The use of strengthening elements is an effective method of improving the mechanical properties of welds, but in laser–arc welding alone, the element distribution is non-uniform, leading to differences in mechanical properties at various positions. Although laser–arc hybrid welding enhances element diffusion due to longer solidification time and higher-speed pool flow, it still cannot ensure uniform distribution of elements in the molten pool, leading to differences in mechanical properties at different positions.

Yan et al. [74] investigated the effect of gravity on weld properties and residual stress by modifying the angle of the base metal (within the range of 0–90°). As per their study results, the microstructure and residual stress were symmetrically distributed only when the weld was vertically inverted. As the angle increased from 0° to 90°, the micro-grain size of the upper side of the weld increased from 66.9 μm to 113.9 μm, which resulted in a decrease in tensile strength from 352.8 MPa to 319.8 MPa. The weld metal flowed to the lower side, resulting in uneven temperature distribution on both sides of the weld. Additionally, non-uniform solidification on both sides of the weld led to asymmetric distribution of longitudinal and transverse residual stress. Numerical simulation can obtain and verify the impact of the laser–arc interaction on the weld seam mechanical properties, making it a cost-effective and simplified alternative to experimental testing.

**Figure 5:**
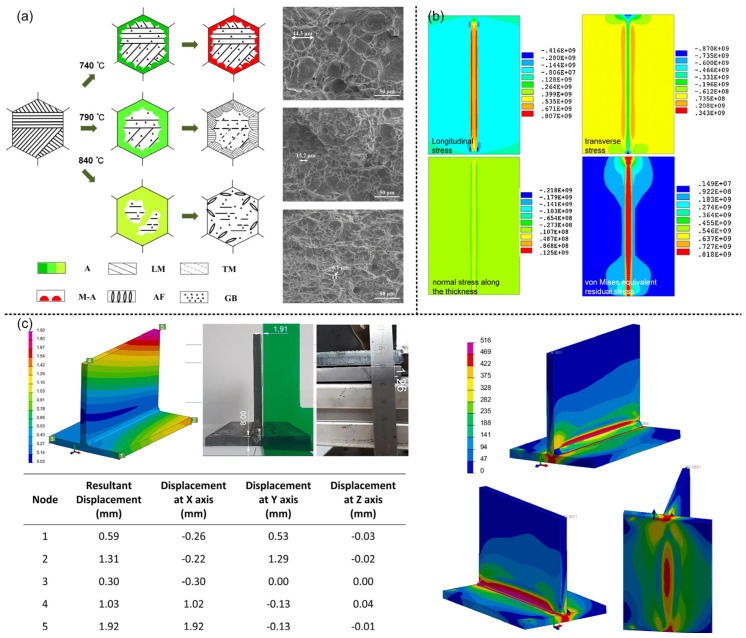
(**a**) Left: Schematic diagram of microstructure transformation at different secondary peak temperatures (A: austenite, LM: granular bainite, TM: tempered martensite, M-A: necklace-type M-A component, AF: acicular ferrite, GB: parent material), right: tensile fracture micrograph [75]; (**b**) stress and residual stress in different directions in hybrid welding [46]; (**c**) left: multi-position deformation in simulation and experiment, right: residual stress distribution in multi-view [76].

Zhan et al. [12] found that the welding time, material consumption, and deformation peak value of MIG were 8, 16, and 3 times those of MIG and laser-MIG hybrid welding, respectively. It was also shown that hybrid welding can achieve smaller heat input with higher filling efficiency and welding speed, and finally achieve smaller welding deformation. Liu et al. [47] concluded that the elasticity of the martensite formed during welding had little impact on residual stress. Furthermore, they found that residual stress increased with higher annealing temperatures. Jiang et al. [55] found that because of the smaller heat input and weld cross-sectional area compared with traditional MIG welding, laser-MIG hybrid welding has great advantages in grain refinement and porosity suppression. The smaller thermal input means the lower temperature gradient and peak temperature of hybrid welding, and there is a close relationship between the solidification rate, phase transformation, and temperature field; therefore, the crystallization and phase transformation in laser–arc hybrid welding deserve our attention. As shown in Figure 5a, Qi et al. [75] studied the effect of secondary peak temperature on the microstructure and the mechanical properties of X100 pipeline steel using a numerical simulation. The impact energy of HAZ increased gradually (the maximum was 37.2 j) and the dimple size at tensile fracture decreased gradually (minimum is 6.1 μm). The increase in the amount of reversion austenite leads to the decrease in the carbon concentration and the formation of different microstructures. As shown in Table 1, the different peak temperatures (740, 790 and 840 °C) corresponded to the microstructures of necklace-type M-A, necklace-type martensite, and granular bainite + acicular ferrite, respectively.

As shown in Figure 5b, by simulating the temperature and stress fields of NV E690 Steel, Sun et al. [46] found that the residual stress near the fusion zone and the heat-affected zone was high and showed tensile stress. As explained, the large grain size in HAZ is the main factor causing the large residual stress; as shown in Figure 5c, Churiaque et al. [76] optimized the parameters of fillet welds of 8 mm-thick EH36 steel plate using a numerical simulation, and showed that the large grain size in HAZ is the main factor causing the larger residual stress. Finally, the full penetration welds with displacement disturbance of 1.29 mm for the bottom plate, 1.92 mm for Rib Plate and 380 MPA for maximum residual stress are obtained, the welding speed (2.2 m/min) is 1.76 times higher than that of Valdaytseva et al. [77] (1.25 m/min). Because of the difference in action point between the laser and arc center in the paraxial coupling, both of them will produce a molten pool and converge, and then the molten pool will be affected by both. The molten pool with shallow and wide arc [78] and the molten pool with narrow and deep laser flow influence each other. Cho et al. [79] found that the liquid in the arc bath can rapidly expand to the laser heating point, which increases the transverse diffusion of the bath, produces a larger front beam angle, and then reduces the stress concentration; under the influence of non-uniform temperature field, the difference between the deformation caused by thermal expansion at different positions and the constraint caused by the thermal expansion will eventually affect the residual stress and mechanical properties in this area. Qian et al. [80] found that in LAHW, the sequence of residual stress from large to small is the fusion line and heat-affected zone, laser zone, arc zone, mixed zone, bottom of weld, and top of weld. Zhang et al. [81] found that the fluid flow has a great influence on the temperature field in the hybrid welding; this was determined based on a numerical simulation of the melt flow near the keyhole of TCS stainless steel.

In laser–arc hybrid welding, because of the wider pool and more complex pool, the solidification time of the pool is much higher than that of single-laser or arc welding. The longer solidification time increases the interaction between laser and arc. The results show that this method can reduce the impact force of droplet on the molten pool, increase the time and velocity of bubble overflow due to keyhole collapse, and reduce the plastic strain and residual stress due to uneven thermal expansion. The reduction in the occurrence probability and the degree of influence on the molten pool can refine the grain structure and strengthen the mechanical properties.

### 4.3. Laser–Arc Hybrid Additive Manufacturing

**Figure 6:**
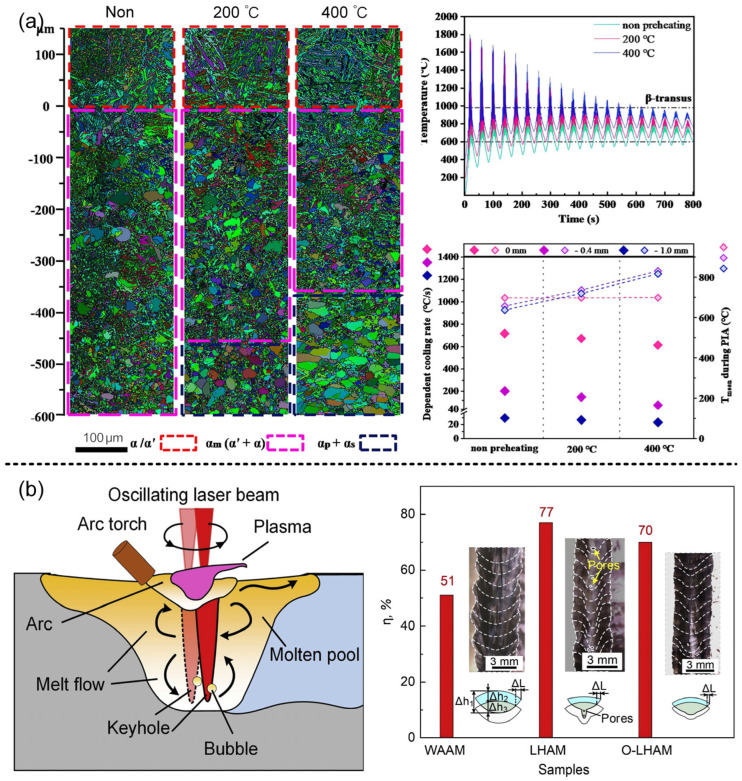
(**a**) microstructure (left) and temperature distribution (right) in the welding region [83]; (**b**) keyhole bubble trapping schematic diagram (left) and cross-sectional morphology under different welding methods (right) [84].

Compared to both laser and arc additive manufacturing, laser–arc hybrid additive manufacturing offers nearly all the advantages of hybrid welding. Its high deposition rate makes it advantageous in the production of large-scale aviation and transportation components. The wider pool area and longer solidification time are beneficial for uniform element distribution and bubble overflow. Complex flow interactions can also effectively enhance pool stability. Liu et al. [82] compared the microstructure and mechanical properties in different regions of arc additive manufacturing and laser–arc hybrid additive manufacturing and found that the periodic changes in the microstructure are the same. The coarse columnar, fine columnar and fine equiaxed grain regions are found at the bottom, middle, and top, respectively, but the grain refinement regions appear in the manufacturing of hybrid materials due to the laser thermal effect. In addition, there are fewer Al-Si-Sr phases and more homogeneous Sr elements in the hybrids. These factors increased the microhardness by 4.2 HV_0.05_ and the tensile strength by 20.8 MPa. In additive manufacturing, preheating has an important effect on the microstructure phase transition, as shown in Figure 6a. Cui et al. [83] performed numerical simulation, and presented the effect of preheating temperature on the microstructure phase transition of materials. When the preheating temperature increases to 400 °C, the welding area decreases by 600 μm compared with that without preheating. The preheating can promote the formation of secondary α phase, which can effectively increase the mechanical properties of the members as a strengthening phase. By optimizing the heat source model, Sun et al. [46] presented the distribution characteristics of residual stress in 7A52 aluminum alloy members welded by laser–arc hybrid welding, and found that the variation of beam height only affected the longitudinal residual stress in the substrate and the beam; it had little effect on the transverse residual stress of the substrate, and the corresponding substrate constraints had little effect on the transverse residual stress of the beam.

Laser–arc hybrid additive manufacturing inherits the benefits of laser–arc hybrid welding, including enhanced deposition efficiency, improved formation, stabilized molten pool, and grain refinement. However, it also inherits and potentially intensifies the negative aspects of hybrid welding, such as the high level of difficulty, post-processing requirements, and internal defects after forming. The introduction of oscillating laser technology offers a new approach to address these limitations. As shown in Figure 6b, Gong et al. [84] introduced laser oscillation into the manufacturing of hybrid additives and studied the influence of an oscillating laser on the manufacturing of hybrid additive. They found that the addition of an oscillating laser has great advantages in stabilizing droplet transition, reducing surface roughness and grain refinement, etc. The oscillating laser–arc hybrid welding also has its great advantages in deposition effect and shaping. As shown in Table 2, the section of arc increasing material is crescent-shaped, while the cross section of the laser–arc hybrid is in the shape of a wine glass. The section shape of the oscillating laser–arc hybrid is between the arc and laser–arc hybrid. Its remelting depth is the smallest, and its remelting rate reaches 70%, which is 1.4 times that of arc-increasing material.

The potential benefits of oscillating laser–arc hybrid additive manufacturing, including improved forming, reduced porosity defects, refined grains, and strengthened properties, are not yet fully understood due to limited research on the internal mechanisms. There is a particular lack of studies on numerical simulation of molten pool flow and element distribution, stress, strain, and residual stress. Further research is needed to fully comprehend this technology.

### 4.4. Oscillating Laser–Arc Hybrid Welding

In oscillating laser welding, it has been proved that the oscillatory behavior of a laser heat source has the advantages of improving forming, suppressing defects, refining grains and strengthening properties because of its special locus of motion [53,85]. Oscillating laser–arc hybrid welding (O-LAHW) has shown excellent metallurgical ability and wide adaptability in many types of material welding due to the advantages of both O-LAHW and arc integration [84,86]. For example, Meng et al. [33] found that weld formation and non-fusion defects in steel/aluminum dissimilar joints can be effectively suppressed at an oscillating frequency of 150 Hz. The physical model and coupling mechanism of the interaction between the oscillating scanning laser and the electric arc can be clearly described by numerical simulation, and its model and mechanism can be optimized and perfected [87].

**Figure 7:**
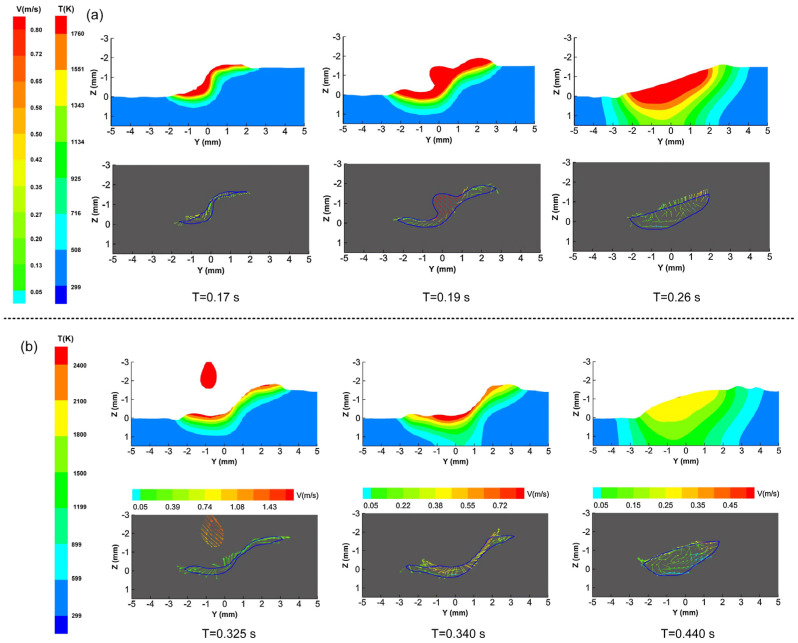
(**a**) The change rule of temperature field and flow field at x = 20 mm; (**b**) the change rule of temperature field and flow field at X = 33 mm [45].

In Gao et al.’s [45] numerical simulation of oscillating scanning laser–arc hybrid welding using double ellipsoidal and cylindrical heat sources on lap plates, it was observed that the width and depth of the weld line increased with the arc droplet’s entry into the weld pool, as shown in Figure 7a. At the moment of droplet contact with the molten pool, the liquid metal flow direction can be divided into up and down, and the sinusoidal oscillation of the laser is uniformly distributed throughout the molten pool. Due to the varying heat transfer between the upper and lower molten pools in oscillating scanning laser–arc hybrid welding, the temperature gradient and heat transfer efficiency of the upper plate is higher than that of the lower plate. As depicted in Figure 7b, at x = 33 mm, the shallow molten pool under droplet impact causes the entire molten flow to divide into two directions. Whilst the sinusoidal oscillating scanning laser produces a larger and more uniform energy and temperature field, the impact angle of the droplet results in greater heat input to the plate. As a result of the combined action of impact force, gravity, and Marangoni force, resultant forces on the upper and lower parts appear in different directions.

**Figure 8:**
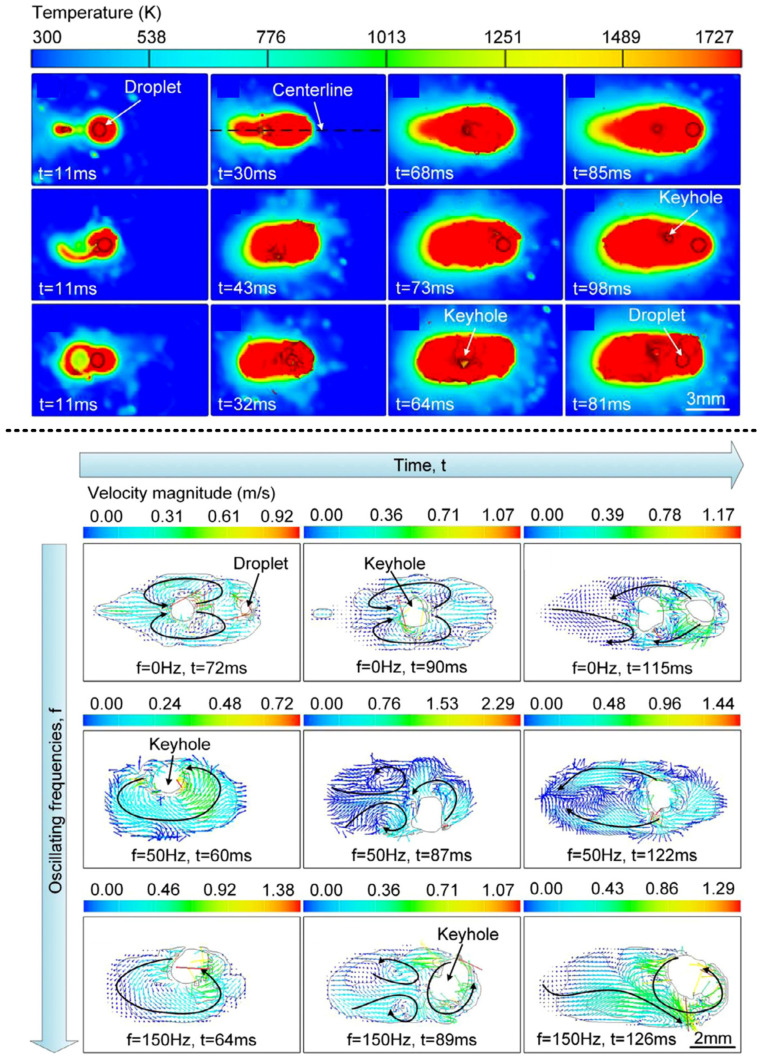
**Top:** The variation in temperature field with time at oscillating frequencies of 0, 50, and 150 Hz; **bottom:** the variation in the flow field with time at oscillating frequencies of 0, 50, and 150 Hz [53].

Shi et al. [53] carried out a numerical analysis of circular oscillating laser–arc hybrid welding of 304SUS stainless steel. As shown in Figure 8a, with the increase in the oscillation frequency, the keyhole depth in the weld pool becomes shallow, the keyhole opening increases, the weld pool becomes shallow, and the weld porosity decreases before disappearing entirely. As shown in Figure 8b, when the oscillation frequency is low, the eddy current direction in the molten pool changes with the change in the laser beam direction. When the laser beam oscillates forward, it forms an anticlockwise vortex, and when the laser beam oscillates backward, it forms a clockwise vortex. Because the oscillating-induced eddy current can significantly improve the temperature and flow fields, circular oscillating laser–arc hybrid welding has a more stable welding process, fewer defects, and a lower weld depth-to-width ratio. When the oscillating frequency is higher than 150 Hz, the eddy current velocity can reach 0.7 ms to control the flow field, stabilize the molten pool, and realize the aim of restraining spatter and porosity.

## 5. Conclusions

In this paper, the effects of laser–arc interaction on weld formation, multi-physical fields (temperature field, flow field, and stress field), stability of welding process, defects, and microstructure and properties are reviewed. The strengthening mechanism and element distribution of different elements in welding wire are widely concerned, but the research on microstructure evolution and solute element distribution in LAHW is lacking. Although there is a lot of literature on the research of oscillating laser welding numerical simulation, there are few types of research on the numerical simulation of O-LAHW, the physical mechanism of the interaction between the oscillating laser and the arc is not perfect, and its effect on the evolution of multi-physical fields, welding defects, microstructure, and mechanical properties is not clear. To enhance the understanding of microstructure strengthening and mass transfer crystallization, future numerical simulations of oscillating laser–arc hybrid welding (O-LAHW) should be conducted. Additionally, the benefits of O-LAHW, including the influence of the oscillating laser on the molten pool, keyhole stability, and performance enhancement, should be explored further. Understanding these key areas of O-LAHW will be critical to the future advancement of this technology.

## Figures and Tables

**Table 1:** Secondary thermal cycle products at austenite grain boundaries at different temperatures [75].

Temperature	740 °C	790 °C	840 °C
Products of the secondary thermal cycle	Necklace-type M-A constituent	Necklace-type lath martensite	Granular bainite + acicular ferrite

**Table 2:** The single-layer penetration depth (Δh_1_) and layer height (Δh_2_) in three increasing materials [84].

	Arc	Laser–Arc Hybrid	Oscillating Laser–Arc Hybrid
Δh_1_ (mm)	4.1	3.5	2.7
Δh_2_ (mm)	2	0.8	0.8

## Data Availability

Not applicable.
